# Activated Oxytocin Neurons in the PVN-DVC Pathway in Asthmatic Rats

**DOI:** 10.3389/fnana.2020.00047

**Published:** 2020-07-31

**Authors:** Zhe Chen, Li Long, Jian Xiao, Nina Liu, Rong Dong

**Affiliations:** ^1^Department of Pulmonary and Critical Care Medicine, Immunology Laboratory, Affiliated Kunshan Hospital of Jiangsu University, Suzhou, China; ^2^Department of Pulmonary and Critical Care Medicine, The Second Affiliated Hospital of Guangzhou Medical University, Guangzhou, China; ^3^Department of Physiology, Medical School, Southeast University, Nanjing, China

**Keywords:** asthma, paraventricular nucleus, dorsal vagal complex, oxytocin, pathway

## Abstract

Asthma is a heterogeneous disease, and the central nervous system (CNS) also participates in the pathogenesis of asthma. We previously reported the amygdala might regulate asthmatic attacks via projecting to the paraventricular hypothalamic nucleus (PVN). The dorsal vagal complex (DVC) is a crucial region that modulates respiratory. This study aimed to observe the activity in both PVN and DVC and the connection between PVN and DVC in asthmatic rats. Immunohistochemistry was conducted to observe the changes in Fos and oxytocin (OT) expression. Retrograde tracing using wheat germ agglutinin-horseradish peroxidase (WGA-HRP) and double immunohistochemistry for OT and Fos was used to observe the HRP/OT/Fos positive neurons distribution in the PVN. The results showed that during an asthma attack, the Fos positive neurons increased in both PVN and DVC over time. The expression of OT positive neurons in PVN showed a similar trend in parallel to the c-Fos positive neurons in PVN. The HRP retrograde-labeled neurons were densely distributed in the medial and lateral subnucleus in the PVN. OT^+^/HRP^+^ and Fos^+^/OT^+^/HRP^+^ accounted for 18.14%, and 2.37% of HRP-labeled neurons, respectively. Our study showed PVN and DVC were activated and the expression of OT positive neurons in PVN were increased over time during an asthma attack. The existence of connection between PVN and DVC suggested the OT neurons in PVN might project to DVC which might be involved in the pathogenesis of asthma.

## Introduction

The pathogenesis of asthma is complex and has not yet been fully clarified. Recent studies have indicated that the central nervous system (CNS) also participated in the initiation of an asthma attack, especially in some patients with emotional disorders (Kline and Rose, 2014; Ritz et al., 2019a). The vagus nerve can transmit peripheral inflammatory information to the brain, which is a crucial pathway for immune-brain communication (Dantzer and Wollman, 2003; Wrona, 2006; Mazzone and Undem, 2016). The hypothalamus, the high center of the nucleus of the solitary tract (NTS), can regulate NTS functional activities, including an essential regulatory effect on breathing (Costello et al., 1991). Peripheral inflammatory information is transmitted to the NTS through the vagus nerve (Simonyan et al., 2012; Chen et al., 2017) and a reciprocal connection occurs between the NTS and the hypothalamus. The paraventricular hypothalamic nucleus (PVN) is also involved in asthma attacks (Costa-Pinto et al., 2005, 2007). During an asthma attack, neuronal activity in the PVN promotes airway hyper-responsiveness by causing intrapulmonary inflammation (Costa-Pinto et al., 2006). Previously, we reported that an asthma attack could excite neurons in the medial amygdala (MeA) and the central amygdala (CeA). This connection of the amygdala and PVN might be correlated with the co-occurrence of asthma (Chen et al., 2020) and oxytocin (OT) in PVN. This connection could play a vital role in the regulation of asthma attacks. The dorsal vagal complex (DVC), including NTS and the dorsal motor nucleus of the vagus (DMV), is a crucial region that modulates respiratory, cardiovascular, and digestive function. In this study, we aimed to explore the activity in both PVN and DVC and the connection between PVN and DVC in asthmatic rats.

## Materials and Methods

### Animals and Models

Male Sprague–Dawley rats (weighing 250–350 g) were fed in a quiet environment at 18–25°C, housed with controlled lighting in a 10:14 h/day, day:night cycle with free access to food and water. The rats were acclimatized to the environment for 1 week before the experiments started. All animal experiments and procedures were performed in accordance with the *Guide for the Care and Use of Laboratory Animals* published by the U.S. National Institutes of Health. As in a previous study (Elwood et al., 1991) ovalbumin (OVA, 100 mg), aluminum hydroxide (100 mg), and a mixed suspension of inactivated *Bordetella pertussis* (5 × 10^9^ copies) were administered intraperitoneally (i.p.) to rats in the experimental group on days 1 and 3 (1 ml each time). On days 15–17, a 1% OVA:saline solution was ultrasonically atomized and administered to the rats for 20 min (2–3 ml/min, particle diameter ≤5 μm). The rats with asthma presented with irritability, polypnea, forced respiration, gasps, evident abdominal muscle shrinkage, and coughs. The control animals received normal saline (NS) injected instead. The injected NS (pH 7.2–7.4) and OVA were maintained at 37°C.

### Pulmonary Function Test

Asthmatic and control rats were anesthetized with 0.4% pentobarbital sodium (40 mg/kg, i.p.). The anal temperature was approximately 36–38°C. The limbs and head were fixated in a supine position. The trachea was separated, and an inverted T-shaped incision was cut in the trachea. A pipe that was connected to an airflow exchanger was inserted. The esophagus was incised transversely, then a water injection catheter with four-side holes was inserted, and a pressure transducer was connected to measure the pressure in the esophagus to maintain the intrathoracic pressure. Respiratory flow and esophageal pressure signals were collected by an RM6240 multi-channel physiological signal acquisition and processing system connected to a computer to record lung function 30 min before and after the asthma attack. The respiratory frequency (RF), tidal volume (V_*T*_), minute ventilation volume (MVV), expiration time/inspiration time (T_E_/T_I_), airway resistance (R_aw_), dynamic pulmonary compliance (C_dyn_), frequency of diaphragmatic electric activity (EMGdi frequency), and integrated diaphragmatic electrical activity (IDEA) were calculated (Zhuang et al., 2015). The signal collection rates, filtering parameters, and magnification were consistent throughout the experiment.

### Hematoxylin and Eosin (H&E) Staining and Bronchoalveolar Lavage Fluid (BALF)

Asthma and control rats were anesthetized with 0.4% sodium pentobarbital solution (40 mg/kg, i.p.). The rat chest cavity was exposed, and the right atrial appendage was incised, the right hilar was ligated, and the right lower lung was collected for histopathological staining. A total of 3 ml of 0.3% phosphate-buffered saline (PBS) solution at pH 7.4 and 37°C was injected into the trachea and recycled five times. Bronchoalveolar lavage fluid (BALF) was collected in a centrifuge tube, incubated in a water bath at 4°C and centrifuged at 1500 rpm at 4°C for 10 min. The pellets were collected for eosinophil and white blood cell counting. The total cell count was counted using a Bürker chamber and the differential cell count was evaluated by hematoxylin and eosin (H&E) staining. Four hundred cells were counted for differential cell count analysis. The left lung tissues were removed for H&E staining.

### Blood Gas Analysis

Asthma and control rats were anesthetized with 0.4% sodium pentobarbital solution (40 mg/kg, i.p.) at 5, 10, or 30 min after the onset of OVA-induced asthma. Tracheal and carotid intubations were performed. Arterial blood was collected for blood gas analysis. The changes in P_a_O_2_, P_a_CO_2_, and S_a_O_2_ were measured.

### Immunohistochemistry

Based on the time dependence of Fos protein expression, the asthmatic rats were divided into groups corresponding to 30, 60, 90, and 120 min after an asthma attack. Sections of the PVN were stained for Fos and OT, and sections of the DVC were stained for Fos expression. Animals were anesthetized by i.p. injection of 0.4% pentobarbital sodium (40 mg/kg), and the heart was exposed by opening the ribcage. The left ventricle was cut, a perfusion needle was inserted into the ascending aorta, and the right atrial appendage was cut. The blood was quickly rinsed with 150–200 ml of NS until the liquid flowing out was colorless. Then, 4% paraformaldehyde (PFA; 400–500 ml, 4°C) was subsequently perfused. After perfusion, the brain tissue was post-fixed in 4% PFA at 4°C for 4 h followed by immersion in 30% sucrose solution at 4°C for 48 h. Coronal slices of the tissues were cut using a frozen microtome (thickness, 30 μm) for immunohistochemistry. Sections were incubated with 3% H_2_O_2_ for 15 min to block endogenous peroxidase activity, washed with 0.3% PBS (3 × 5 min), incubated for 1 h at room temperature with a blocking solution (10% goat serum), and incubated overnight with primary antibody (rabbit anti-Fos; 1:500; Santa Cruz, or rabbit anti-OT; 1:1000; Millipore). The tissue was washed with 0.3% PBS (3 × 5 min), followed by incubation for 1 h at room temperature with a biotinylated secondary antibody (goat anti-rabbit; 1:300; Abcam). After washing with 0.3% PBS (3 × 5 min), sections were incubated for 30 min with horseradish peroxidase (HRP) and washed with 0.3% PBS (3 × 5 min) before reacting with diaminobenzidine (DAB) as a chromogen. Sections were examined using an Olympus light microscope.

### Wheat Germ Agglutinin-Horseradish Peroxidase (WGA-HRP) Retrograde Tracing

Asthma rats were anesthetized by i.p. injection 0.4% of pentobarbital sodium solution (40 mg/kg) and located at DVC according to the Paxinos and Watson rat brain map. The latch was set as the reference point. The rats were then fixed into a stereotaxic frame, and the scalp was shaved and disinfected. An incision was made along the midline of the skull, and the muscles of the neck were separated bluntly to expose the region of interest. The posterior membrane was cut to reveal the back of the medulla. Under stereotaxic guidance, an opening of 0.3–0.5 mm was made next to the midline with a depth of 0.8–1.0 mm. Then, 0.1 μl of 4% Wheat germ agglutinin-horseradish peroxidase (WGA-HRP) was slowly injected into one side of the DVC using a micro-glass tube. The injection was performed at a constant rate (0.014 μl/min) with a microinjector for approximately 7 min. The microinjector was then removed, and the muscle and the skin were sutured and disinfected. Rats were individually housed for 48 h before brain tissue extraction.

Tetramethyl benzidine (TMB) slices were prepared through a WGA-HRP color reaction in accordance with the TMB method described by Mesulam (1978). The slices were collected in 0.1 mol/l phosphate buffer (pH 7.2–7.4), rinsed in distilled water three times for 2–3 min, dipped in a TMB reaction solution for 20 min to be protected from light, and incubated in the dark at 15–20°C. Afterward, 0.7 ml of 0.3% H_2_O_2_ was added to each 100 ml of TMB reaction solution every 10 min. Color development was carefully monitored under a microscope and terminated by washing with a detergent solution diluted in 0.2 mol/l PB (pH 5.0–5.4) at a 1:4 ratio 3–6 times for 2–4 min. The patch was air-dried and counterstained with neutral red to show the cell morphology, locate the injection site, and observe the distribution of retrograde-labeled neurons. The slices were placed in double-distilled water for 30 s and transferred to 1% neutral red dye solution for 5 min. Gradient alcohol dehydration was performed using 70 and 95% ethanol twice for 30 s, then absolute ethanol three times for 3 min. Finally, the samples were immersed in xylene twice for 3 min each, and a neutral gum seal was added after sections became transparent.

### Triple-Labeled Staining of WGA-HRP Retrograde Tracing Combined With Fos and OT Immunohistochemical Staining

For triple staining, WGA-HRP stained tissues were then stained for Fos and OT. In Fos staining, a blue DAB chromogenic reagent was used; to stain for OT neurons, a yellow DAB chromogenic agent was used. For these staining reactions, tissues were prepared by washing with 0.01 mol/l PBS four times. The reaction was carefully monitored with a microscope and terminated with distilled water. Gradient alcohol dehydration was performed using 70 and 95% ethanol twice for 30 s, followed by absolute ethanol three times for 3 min. Finally, the samples were immersed in xylene for 3 min twice, and a neutral gum seal was added after sections became transparent.

### Statistical Analysis

Data were expressed as the mean ± standard deviation and analyzed for significant differences using SPSS 17.0 software. The mean density of immunohistochemistry was determined using Image-Pro Plus (IPP). In the process of measuring the density of staining, the experimental parameters are consistent to detect the average density value. Six sections in each brain tissue were selected randomly for statistical analysis. Comparisons among multiple groups were performed using one-way analysis of variance (ANOVA), and Student’s *t*-test was used for comparison of two groups. A *p*-value <0.05 was considered statistically significant.

## Results

### Changes in Pulmonary Function and Airway Inflammation in Asthmatic Rats

The asthmatic rats were subjected to 1% OVA inhalation on days 15–17 after they were sensitized to stimulate asthma attack. After 5 min of inhalation, asthma symptoms, such as restlessness, scratching ears and cheeks, shortness of breath, visible contraction of abdominal muscles, nodding breath, and wheezing were observed in the rats. These symptoms peaked at approximately 15 min and lasted 30 min. Cyanosis and limp limbs occasionally occurred in severe cases.

**FIGURE 1:**
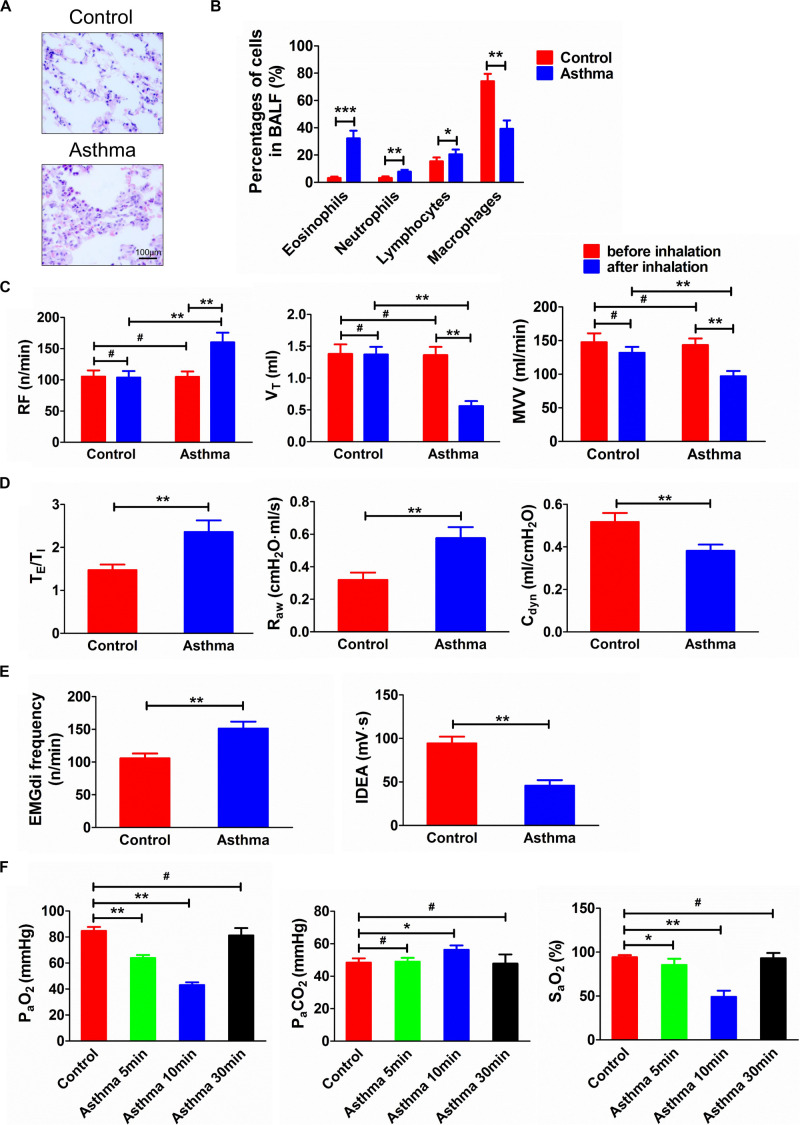
**(A)** Histopathological changes of lung tissues in asthma and control rats. **(B)** Percentage of cells in BALF in asthma and control rats. **(C)** Changes of RF, V_T_, and MVV before and after challenge. **(D)** T_E_/T_I_, R_aw_, and C_dyn_ in asthma and control rats. **(E)** EMGdi frequency and IDEA in asthma and control rats. **(F)** Changes of P_a_O_2_, P_a_CO_2_, and S_a_O_2_ in control and asthma (5, 10, and 30 min) rats. After the rats were challenged, the lung function was deteriorated and the relevant indicators of ventilation function were significantly changed. Meanwhile, the airway inflammation, especially the eosinophils in BALF, was significantly increased. At the same time, as the duration of an asthma attack increased, arterial oxygen saturation was decreased. *p<0.05, **p<0.01, ***p<0.001, and #*p*>0.05, respectively. *n* = 6 per group. BALF, bronchoalveolar lavage fluid; RF, respiratory frequency; V_T_, tidal volume; MVV, minute ventilation volume; T_E_/T_I_, expiratory time course/inspiratory time course ratio; R_aw_, airway resistance; C_dyn_, dynamic pulmonary compliance; EMGdi frequency, frequency of diaphragmatic electric activity; IDEA, integrated diaphragmatic electrical activity.

Inflammatory cell infiltration could be observed in the bronchial wall and pulmonary interstitial space in the asthmatic rats, and the proportion of eosinophils in BALF increased significantly (*p* < 0.001) (Figures 1A,B). In the control rats, the bronchial, alveolar, and pulmonary interstitial structures of the lung tissue were intact, and the epithelium was smooth.

After the asthma attack, the RF increased (*p* < 0.01), and the V_T_ and ventilation volume per minute of rats decreased significantly (*p* < 0.01). The rat breath was shallow and fast, lasting approximately 30 min. Compared with the control rats, the frequency of diaphragmatic electric activity, the ratio of expiratory:inspiratory time, and R_aw_ increased (*p* < 0.01). However, the integral amplitude of phrenic discharge and C_dyn_ decreased (*p* < 0.01) (Figures 1C–E).

Compared with the control group, P_a_O_2_ at 5 min and 10 min decreased significantly (*p* < 0.01), while S_a_O_2_ at 5 and 10 min decreased significantly (*p* < 0.05 and *p* < 0.01, respectively). However, P_a_CO_2_ showed a significant increase only at 10 min after an asthma attack (*p* < 0.05) (Figure 1F).

### Fos Immunoreactive Substance in the PVN and DVC in Asthmatic Rats

**FIGURE 2:**
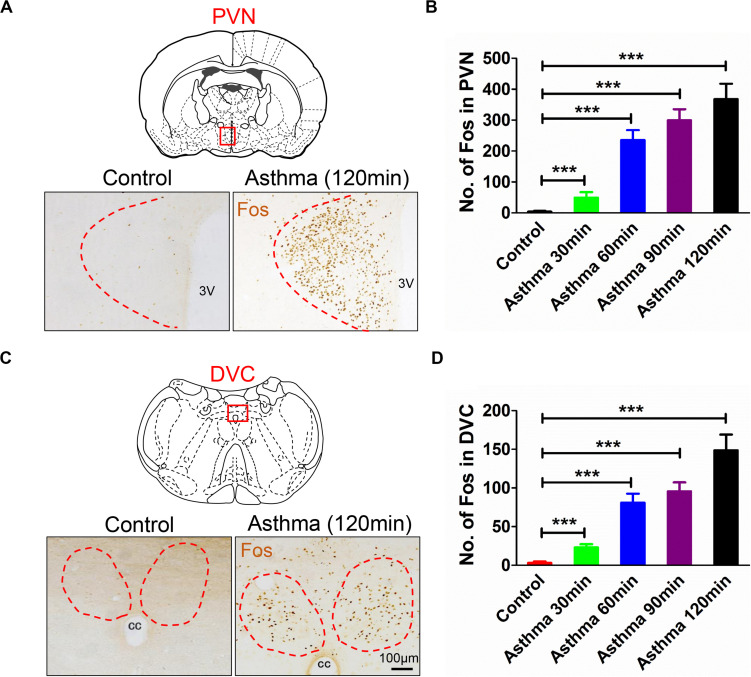
**(A)** Fos expressions in PVN in asthma and control rats. **(B)** Counts of Fos-positive neurons in PVN in control and asthma (30, 60, 90, and 120 min) rats. **(C)** Fos expressions in DVC in asthma and control rats. **(D)** Counts of Fos-positive neurons in DVC in control and asthma (30, 60, 90, and 120 min) rats. The nucleus of the Fos-positive neurons was brownish yellow staining, and Fos expressions increased gradually in the 30, 60, and 90 min and peaked at the 120 min. ***p<0.001. *n* = 6 per group. PVN, paraventricular nucleus; DVC, dorsal vagal complex; 3V, third ventricle; cc, central canal.

The nuclei of Fos-positive neurons were stained brownish-yellow. Following the onset of asthma, the PVN and the DVC were filled with Fos-positive neurons (Figures 2A–C). The Fos immunoreactive substance in the PVN was bilaterally distributed. Compared with the control group, the number of Fos-positive cells increased gradually at 30, 60, and 90 min and peaked at 120 min (*p* < 0.001) (Figures 2B–D). Fos-positive neurons in the DVC were densely distributed in the NTS and DMV, but the density was less than that of Fos-positive neurons in the PVN.

### OT Immunoreactive Substance in the PVN in Asthmatic Rats

**FIGURE 3:**
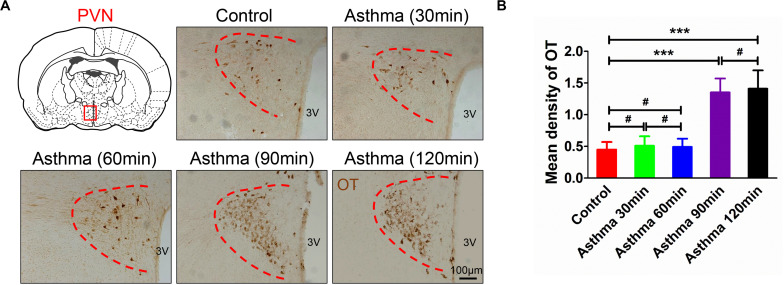
**(A)** OT immunoreactive substance in PVN in asthma and control rats. **(B)** Mean density of OT immunoreactive substance in PVN in control and asthma (30, 60, 90, and 120 min) rats. The OT immunoreactive substance was brown staining, and OT expressions increased and peaked in the 90 and 120 min. ***p<0.001, and #*p*>0.05, respectively. *n* = 6 per group. PVN, paraventricular nucleus; 3V, third ventricle.

Oxytocin-positive neurons were stained brown and primarily distributed in the PVN (Figure 3A). Compared with the control group, there were no differences in the mean density of OT immunoreactive substance among the control rats at 30 and 60 min. However, OT immunoreactive substance increased and peaked at 90 and 120 min (*p* < 0.001) (Figure 3B).

### Distribution of WGA-HRP Retrograde-Labeled Neurons in the PVN

**FIGURE 4:**
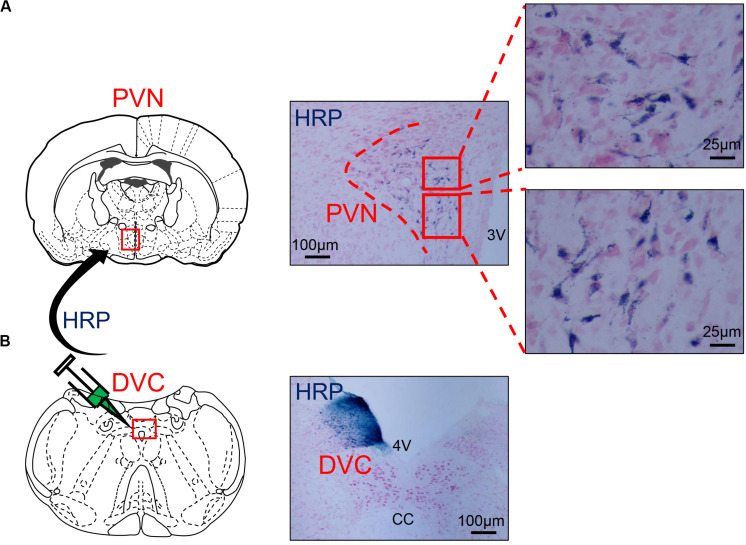
**(A)** The distribution of HRP-labeled neurons in the PVN. **(B)** The microinjection zone of HRP in DVC. HRP injection zone could be observed on one side of DVC, and HRP retrograde-labeled neurons were densely distributed in the medial subnucleus and the lateral subnucleus of PVN and scattered in the dorsal subnucleus. PVN, paraventricular nucleus; DVC, dorsal vagal complex; 3V, third ventricle; 4V, fourth ventricle; cc, central canal; HRP, horseradish peroxidase.

Wheat germ agglutinin-horseradish peroxidase blue reactant could be observed in the WGA-HRP injection zone on one side of the DVC under a light microscope (Figure 4B). HRP retrograde-labeled neurons were densely distributed in the medial subnucleus and the lateral subnucleus of the PVN and scattered in the dorsal subnucleus (Figure 4A).

### Triple-Labeled HRP/Fos/OT-Positive Neuron Distribution in the PVN

**FIGURE 5:**
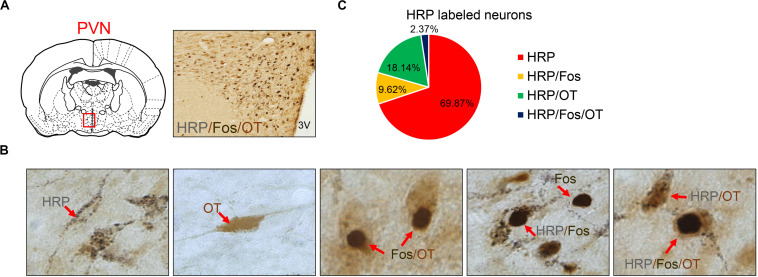
**(A)** HRP/Fos/OT labeled neurons in PVN. **(B)** Seven different immuno-positive neurons (red arrow) distribution in PVN in asthma rats. **(C)** The ratio of different types of HRP labeled neurons. OT labeled neurons, Fos labeled neurons, HRP labeled neurons, HRP/OT labeled neurons, HRP/Fos labeled neurons, Fos/OT labeled neurons, and HRP/Fos/OT labeled neurons were observed in PVN. HRP, horseradish peroxidase; PVN, paraventricular nucleus.

Seven different immuno-positive neurons were observed in hypothalamic slices (Figures 5A,B). Many fine black particles were observed in the cytoplasm of HRP retrograde-labeled neurons. The contour of cell bodies and processes could be observed in some neurons, and the nuclei were not stained. The cytoplasm of OT-positive neurons was uniformly brownish-yellow, the cell bodies and processes were clear, and the nucleus was not stained. The nuclei of Fos-positive neurons were stained brown, the cytoplasm was not stained, and cell bodies and processes were not exposed. HRP/OT double-labeled neurons were brownish-yellow with many black particles in the cytoplasm. Fos/OT double-labeled neurons were brownish-yellow with a black nucleus in the cytoplasm. HRP/Fos double-labeled neurons had a black nucleus surrounded by several black particles. HRP/Fos/OT triple-labeled neurons were brownish-yellow with a large round or elliptical black nucleus and many small black HRP-reactive particles in the cytoplasm.

Eight asthmatic rats were used for WGA-HRP retrograde tracing and double immunohistochemistry. After the average number of the neurons labeled with six random slices of the PVN per rat, the following findings were obtained (Figure 5C). A total of 634 HRP-labeled neurons in the PVN were observed, including HRP (443), HRP/Fos (61), HRP/OT (115), and HRP/Fos/OT (15); these accounted for 69.87, 9.62, 18.14, and 2.37% of HRP-labeled neurons, respectively. The ratio of (HRP/Fos/OT)/(HRP/OT) accounted for 11.6%, indicating that approximately one-tenth of the activated OT neurons in PVN composed PVN-DVC circuit. The HRP/OT and HRP/Fos/OT accounted for 20.51% of the HRP-label neurons, indicating that approximately one-fifth of the PVN neurons that innervated the DVC were OT neurons.

## Discussion

Asthma is characterized by chronic airway inflammation and airway hyper-responsiveness. Three main mechanisms have been proposed to cause airway inflammation in asthma: type I allergic reaction dominated by Th_2_ cytology, i.e., immunogenic inflammatory theory; epithelial injury and reactivation of epithelial–mesenchymal trophic units dominated by transfer growth factor, endothelin-1, and metalloproteinase; and neuropeptide-induced neurogenic inflammatory theory; however, these sources of inflammation cannot fully explain the pathogenesis of various types of asthma (Rogers, 2001). Theories of neurogenic and immunogenic inflammation mainly focus on the study of local factors. When asthma attacks occur, the activity of neurons in multiple nuclei in the brain is enhanced, indicating that the CNS participates in the regulation of asthma (Gao et al., 2019; Ritz et al., 2019b). The CNS may regulate these networks by its control of the neuroendocrine system.

In this study, DVC and PVN neurons were activated following OVA challenge. Fos staining is often used as marker of excitatory morphological localization of neurons in the brain. Fos protein is the expression product of the *c-fos* proto-oncogene, which is an immediate-early gene. It can respond to and express the input information of external stimuli, such as neurotransmitters, hormones, and nerve impulses. In the field of respiratory research, Fos expression could reflect neuronal activation in different conditions (Wakai et al., 2015).

Paraventricular hypothalamic nucleus neurons mainly synthesize and secrete oxytocin and arginine vasopressin (AVP) and produce other transmitters (Japundžić-Žigon et al., 2020) such as enkephalin, corticotropin-releasing hormone, thyrotropin-releasing hormone, and cholecystokinin. The central AVP release may be involved in the stress-induced augmentation of airway vagal activity, and, consequently, the induction or exacerbation of some airway diseases (Yan et al., 2017). OT could regulate respiratory and cardiovascular function (Granjeiro et al., 2014) and may act as a potential cardioprotective peptide and improve tachypnea induced by endotoxemia (Elorza-Ávila et al., 2017). OT in the PVN can regulate a series of neuroendocrine-immune and social functions. Activation of the OT system can promote social behavior (Resendez et al., 2020) and may also be involved in aggressive behavior (Shabalova et al., 2020). The medial subnucleus of the PVN is involved in immune regulation. Following intraventricular injection of IL-1β, expression of Fos was observed in anterior and medial large cell OT neurons of the PVN, indicating that OT neurons in the PVN innervating the medulla oblongata during the immune response were excited (Yang et al., 1997). OT neurons can indirectly regulate immune activity by regulating the activity of autonomic ganglion neurons in the brainstem (Rivier and Shen, 1994). Therefore, OT neurons may also regulate immune function through the autonomic nervous system and inhibit peripheral cellular immunity and humoral immunity. Other studies have shown that elevated levels of OT in peripheral blood may interact with OT receptors on thymocytes and some lymphocyte subsets in the peripheral blood to regulate the functions of these immune cells (Martens et al., 1998). OT in the PVN and peripheral blood may increase in central and peripheral immune responses, and OT may further affect immune function. Asthma is a systemic immune response mediated by IgE. OT may mediate the occurrence and development of asthma by influencing immune function.

**FIGURE 6:**
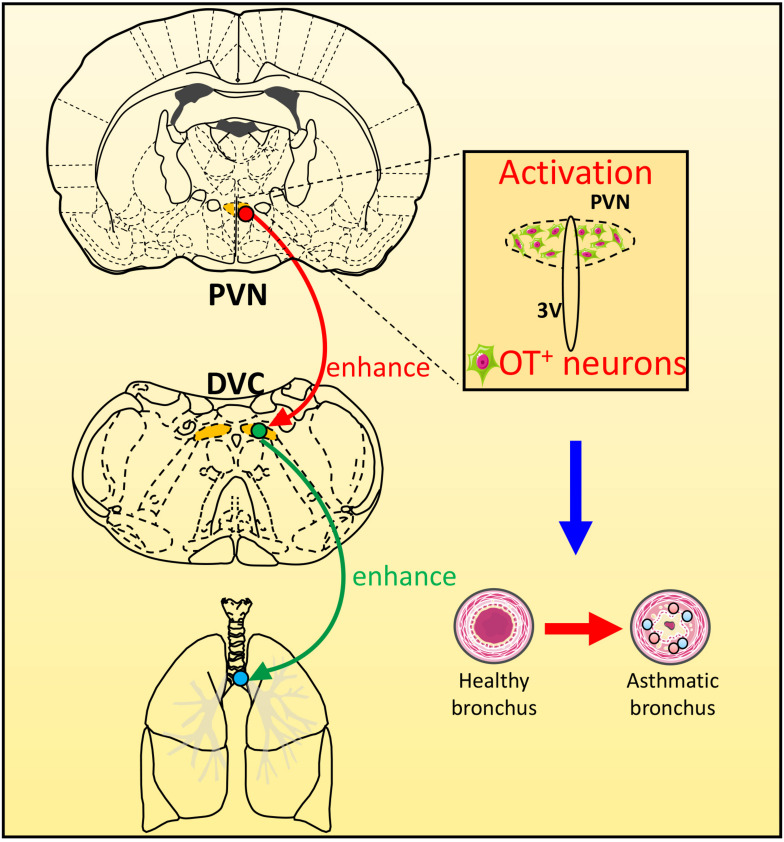
The scheme of the possible role of PVN-DVC circuit on modulating respiratory response during an asthma attack. During asthma attack, neurons in PVN and DVC were activated, and OT expressions in PVN were increased. The OT neurons in PVN may project to DVC to regulate the asthma attack.

In this experiment, WGA-HRP was injected into one side of the DVC to retrograde-label neurons in the PVN. HRP-positive cells were mainly found in the ipsilateral PVN medial subnucleus and the lateral and dorsal subnuclei, which confirmed that neurons in PVN emitted fibers to the DVC. Morphological studies have shown that OT neurons in the medial and dorsal small cell regions of the PVN can be directly projected to the NTS in the dorsal respiratory group (Blevins et al., 2004). The increased excitability of the PVN-NTS pathway may increase the release of OT from the dorsal brainstem (Jackson et al., 2005). Neurotoxin damage of the PVN decreased the OT levels in the dorsal brainstem (Callahan et al., 1992). Sawchenko and Swanson, 1982 reported that OT neurons in the PVN could issue fibers to dominate the DVC. Therefore, we used a triple-label technique combining nerve tract-tracing and double immunohistochemistry to explore the neurotransmitters involved in the PVN-DVC nerve pathway in asthmatic rats (Figure 6). Microscopically, HRP/Fos/OT triple-labeled positive neurons in PVN indicated that OT in PVN might be involved in asthma attack via the PVN-DVC pathway.

Limited by the experimental conditions, we have not tested the effects of central microinjection of an OT antagonist on the intensity and duration of the asthma reaction of OVA inhalation. Further studies will need to examine the effects of activation/inhibition of OT neurons in the PVN-DVC pathway on the parameters of the asthma attack.

## Conclusion

Our study showed PVN and DVC were activated and the expression of OT positive neurons in PVN were increased over time during an asthma attack,. The existence of connection between PVN and DVC suggested the OT neurons in PVN might project to DVC which might be involved in the pathogenesis of asthma.

## Data Availability Statement

All datasets presented in this study are included in the article/supplementary material.

## Ethics Statement

The animal study was reviewed and approved by the Animal Research Committee of Southeast University.

## Author Contributions

ZC, LL, and JX contributed to all aspects of the research. LL and NL contributed to data acquisition, analyses, and interpretation. JX and NL contributed to data acquisition and interpretation. ZC and RD contributed to experimental design, manuscript preparation, and obtained funding for the study. All authors contributed to the article and approved the submitted version.

## Conflict of Interest

The authors declare that the research was conducted in the absence of any commercial or financial relationships that could be construed as a potential conflict of interest.
